# *Rhoifolin* Improves Glycometabolic Control in Streptozotocin-Induced Diabetic Rats by Up-Regulating the Expression of Insulin Signaling Proteins and Down-Regulating the MAPK/JNK Pathway

**DOI:** 10.3390/ph18030361

**Published:** 2025-03-02

**Authors:** Maryam Ehsan, Sibtain Ahmed, Wafa Majeed, Asra Iftikhar, Maryam Iftikhar, Mateen Abbas, Tahir Mehmood

**Affiliations:** 1 Institute of Physiology and Pharmacology, University of Agriculture Faisalabad, Faisalabad 38000, Pakistan; maryameh98z@gmail.com; 2 Department of Biochemistry, Bahauddin Zakariya University (BZU), Multan 60800, Pakistan; 3 Department of Pharmacy, University of Agriculture Faisalabad, Faisalabad 38000, Pakistan; asra7fatima@gmail.com; 4 Akhtar Saeed College of Pharmacy, Rawalpindi 46220, Pakistan; 5 Department of Food Science and Technology, Government College Women University Faisalabad, Faisalabad 38000, Pakistan; 6 Quality Operations Laboratory, Institute of Microbiology, University of Veterinary and Animal Sciences, UVAS, Lahore 63100, Pakistan; mateen.abbas@uvas.edu.pk; 7 Institute of Microbiology and Molecular Genetics (IMMG), University of the Punjab, Lahore 54590, Pakistan; tahir.mmg@pu.edu.pk

**Keywords:** diabetes mellitus, *rhoifolin*, MAPK-8, HOMA-IR, glucose homeostasis

## Abstract

**Background and Aim:** *Rhoifolin* is a bioactive flavonoid that possesses strong antioxidant and anti-inflammatory activities. The current investigation aimed to examine the anti-diabetic potential of *rhoifolin* in streptozotocin-induced diabetic rats. Dose-dependent (10 and 20 mg/kg) anti-hyperglycemic, anti-hyperlipidemic, anti-inflammatory, and antioxidant effects of *rhoifolin* were evaluated by measuring fasting blood glucose, serum glucose, serum insulin, HOMA-IR, lipidemic status, inflammatory cytokines, and hepatic antioxidant markers. To identify the underlying mechanism behind the anti-diabetic activity of *rhoifolin*, qRT-PCR was carried out using rat pancreatic and hepatic tissues. **Results:** The results have shown that *rhoifolin* produced antioxidant effects, as exhibited by DPPH and ABTS^+^ assays, respectively. *Rhoifolin* showed potent alpha-amylase and alpha-glucosidase inhibitory activities. *Rhoifolin* enhanced the serum insulin level, significantly decreased the serum glucose, HOMA-IR, and cytokine levels, and improved the lipid profile. *Rhoifolin* also showed a substantial decline in insulin resistance in the treated rats. *Rhoifolin* significantly raised catalase and superoxide dismutase levels in hepatic tissues while potentially decreasing the malondialdehyde levels. Moreover, *rhoifolin* significantly down-regulated the MAPK-8, TRAF-6, and TRAF-4 expressions and up-regulated the PDX-1, SIRT-1, INS-1, and GLUT-4 expressions in treated groups. **Conclusions:** Our results indicate that *rhoifolin* exhibits a hypoglycemic effect, which appears to be associated with its regulatory impact on metabolic inflammation and oxidative stress markers. This was accompanied by a lower HOMA-IR index, highlighting its potential role in promoting glucose homeostasis and mitigating insulin resistance. According to preliminary results, *rhoifolin* could further be tested to introduce it as another viable treatment option for diabetes.

## 1. Introduction

Diabetes is a persistent metabolic disorder associated with several complications, including hyperglycemia, retinopathy, nephropathy, neuropathy, and cardiomyopathy [1,2]. In 2021, diabetes mellitus affected 10.5% of the population globally, which is estimated to rise to 12% by the year 2045 [3]. Type 1 diabetes mellitus (T1DM) and type 2 diabetes mellitus (T2DM) are marked by elevated blood glucose levels. TIDM results from the destruction of pancreatic beta cells, leading to insulin deficiency, whereas, T2DM, the most frequent form, accounting for 90% of diabetic cases [4], is associated with beta cell dysfunction and insulin resistance. Chronic high blood sugar levels lead to the production of reactive oxygen species (ROS), which interfere with the insulin signaling pathway [5].

At the molecular level, T2DM is manifested by the activation of serine kinase cascades due to hyperglycemia and ROS that dysregulate the insulin signaling mechanism in the phosphatidylinositol 3-kinase/AKT (P13K/AKT) pathway, which is implicated in insulin secretion and glucose transfer. Insulin receptors activated by the PI3K/AKT pathway promote the stimulation of glucose transporter-4 (GLUT-4) in the pancreas, which is a main glucose transporter protein. Increased ROS suppress GLUT-4 levels and exacerbate the tissue’s hyperglycemia and hyperinsulinemia, which cause desensitization of peripheral tissues to insulin [6]. Pancreatic and duodenal homeobox-1 (PDX-1) is a key transcription factor that is important for the development of β-cell function, and glucose-stimulated insulin secretion. Lack of PDX-1 due to the increased level of ROS ultimately inhibits insulin production and exacerbates diabetes [7]. Sirtuin 1 (SIRT-1) is a key modulator of insulin signaling through the activation of AKT, which stimulates insulin receptor substrates. SIRT-1 regulates the release of insulin and protects beta cells from oxidative stress and inflammation-mediated tissue damage [5,8].

Persistent hyperglycemia leads to the progression of diabetic complications, possibly due to the development of cytokines and ROS. Under oxidative stress, the anti-oxidant defence system is overwhelmed, thus causing damage to tissues. Previous literature has revealed that the c-Jun N-terminal kinase (JNK) cascade is activated due to the production of ROS, which has an important role in the progression of diabetes. Oxidative stress and inflammatory mediators impair insulin signaling, alter glucose uptake, and increase gluconeogenesis [7].

Since antiquity, various medicinal plants have been used in Ayurveda and traditional medicinal fields. Such medicinal plants are rich sources of phenolics, flavonoids, terpenoids, coumarins, and other components that show antioxidant, antioxidant, antiapoptotic and hypoglycemic activities [9]. The ability of these plants to preserve the function of pancreatic islet tissues by scavenging free radicals, increasing insulin production, limiting the digestion of carbohydrates and absorption of glucose from the intestines, based on α-amylase and α-glucosidase inhibitory activities, or promoting metabolites in insulin-dependent systems, is attributed to their anti-hyperglycemic effects [10]. In contrast, synthetic anti-diabetic drugs derived from certain compounds exhibit potential side effects [11]. Flavonoids are secondary metabolites of plants, fruits and seeds. Flavonoids contain phenolic hydroxyl groups that mediate their anti-oxidant and anti-inflammatory properties [12].

*Rhoifolin*, also known as rhoifoloside, is a well-recognized tri-substituted flavone (flavonoid) that belongs to the apigenin family. *Rhoifolin*, first extracted from *Rhus succedanea*, is crucial in lowering blood glucose levels. It is also present in numerous dietary sources, such as bergamot, bitter orange, lemon, grapefruit, tomatoes, lupinus, bananas, lablab beans, artichoke, and grapes [13]. *Rhoifolin* has been revealed to own anti-inflammatory, hepato-protective, anticancer and antioxidant activities [14]. It also defends against numerous pathological situations, like osteoarthritis, osteoporosis, neurodegenerative disorders and cardiac dysfunction, by reducing the expression of inflammatory markers and oxidative stress. Furthermore, it helps the immune function recover and displays anti-bacterial and wound healing activities [15].

Metformin is considered a first-line oral synthetic medication for T2DM [16]. It uses a number of methods to produce its anti-diabetic effects [17,18]. Metformin is used as a standard for comparing the anti-diabetic potential of plant-derived compounds because of its established efficacy and safety profile. The present study sought to evaluate the anti-hyperglycemic effect of *rhoifolin* using five groups of rats (normal control, diabetic control, metformin, 10 mg/kg and 20 mg/kg *rhoifolin*-treated groups). The purpose of the normal control group was to establish the base line for comparison, and the diabetic control group was used to determine the progression of diabetes throughout the experimental period. The metformin-treated group was used as the standard for comparing the anti-diabetic effect produced by graded doses of *rhoifolin* in streptozotocin (STZ)-induced diabetic rats.

## 2. Results

### 2.1. In Vitro Antioxidant Activity of Rhoifolin

**Figure 1:**
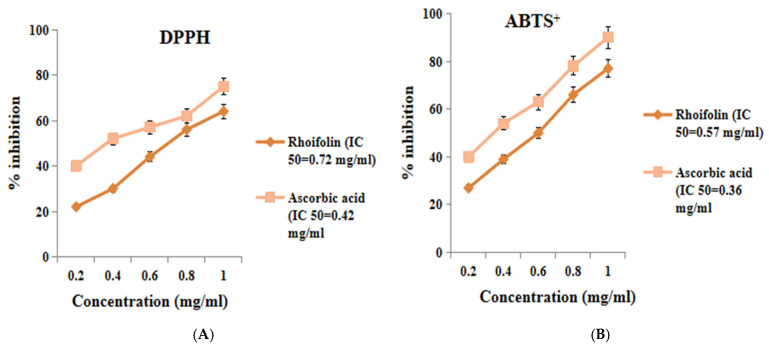
(**A**) DPPH radical-scavenging potential of *rhoifolin*; (**B**) ABTS radical-scavenging activity of *rhoifolin*.

An in vitro antioxidant experiment assessed *rhoifolin*’s capacity to scavenge free radicals. At a maximal intensity of 1 mg/mL, *rhoifolin* exhibited 60% inhibition of the DPPH free radical activity with an inhibitory concentration 50 (IC_50_) of 0.72 mg/mL, compared to ascorbic acid (standard), which demonstrated significantly high % inhibition (74.6%) with an IC_50_ of 0.42 mg/mL. The radical-scavenging potency of *rhoifolin* at a 1 mg/mL concentration to neutralize the ABTS^+^ radicals was 77% with an IC50 value of 0.57 mg/mL (Figure 1).

### 2.2. In Vitro α-Amylase and α-Glucosidase Inhibitory Effects of Rhoifolin

In vitro inhibitory activity of *rhoifolin* for α-amylase and α-glucosidase was assessed at several concentrations between 0.2 and 1 mg/mL. *Rhoifolin* showed the highest α-amylase and α-glucosidase inhibitory tendency at 1 mg/mL conc. in a dose-dependent manner (*p* < 0.05). At a maximal conc. of 1 mg/mL, *rhoifolin* exhibited α-amylase inhibition by 65.7% with IC_50_ 0.58 mg/mL compared to acarbose (IC_50_ = 0.35 mg/mL). *Rhoifolin* also showed 68.4% α-glucosidase inhibition with IC_50_ 0.54 mg/mL compared to acarbose, which exhibited 81.5% inhibition at a maximum conc. of 1 mg/mL with IC50 0.37 mg/mL (Table 1).

### 2.3. Effect of Rhoifolin on Fasting Blood Glucose

**Figure 2:**
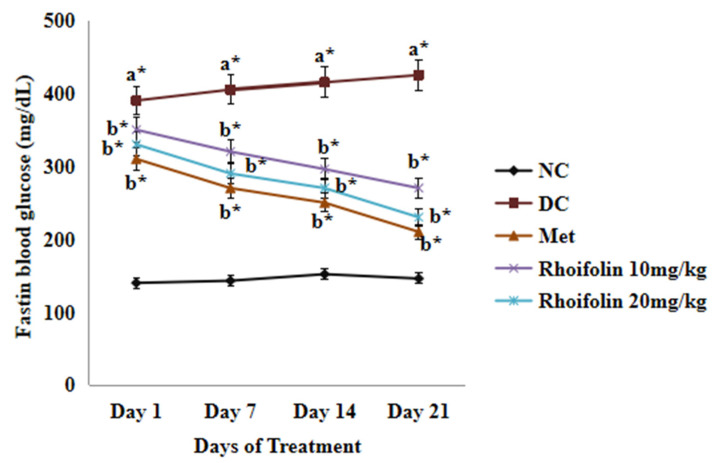
Effect of *rhoifolin* on FBG in diabetic rats on 1st, 7th, 14th and 21st days. Data are represented as Mean ± SD (n = 6/group). Values are significant at ^a^*; *p* < 0.05 represents normal vs. diabetic; ^b^* *p* < 0.05; diabetic control vs. treated groups. (NC: normal control/non-diabetic control; DC: Diabetic control, Met: Metforfin-treated).

Diabetic rats exhibited significantly higher fasting blood glucose (FBG) levels (*p* < 0.05) than the normal control (NC) group rats following streptozotocin administration. However, FBG levels of metformin and *rhoifolin*-treated groups dropped gradually from day 1 to day 21 of the experiment (Figure 2).

### 2.4. Effect of Rhoifolin on Serum Glycometabolic Markers

**Figure 3:**
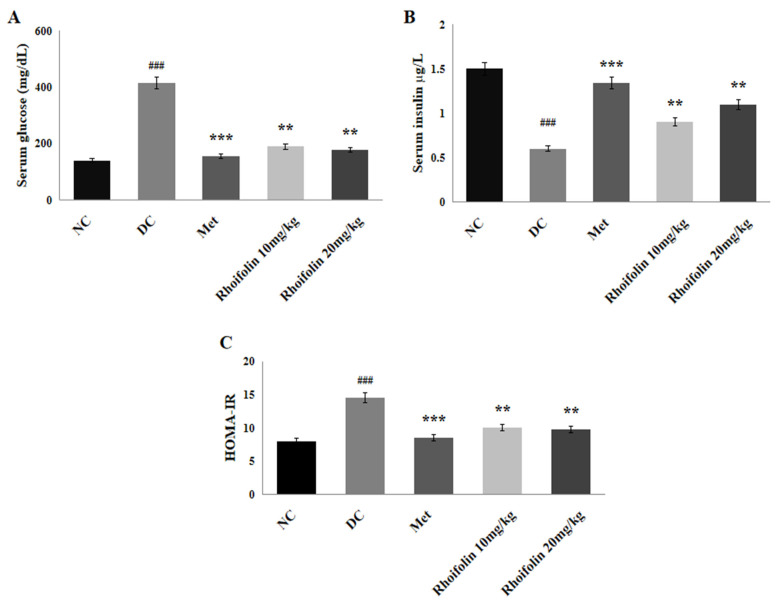
Effects of *rhoifolin* on (**A**) serum glucose; (**B**) serum insulin; (**C**) HOMA-IR. Results are displayed as Mean ± SD (n = 6/group). ^###^ *p* < 0.001 represents normal vs. diabetic; ** *p* < 0.01, *** *p* < 0.001; diabetic control group vs. treated groups.

The results have demonstrated that serum glucose was markedly (*p* < 0.001) augmented in all groups after streptozotocin (STZ) injection compared to the non-diabetic control (NC) group. However, the administration of metformin and graded dosages of *rhoifolin* for 21 days significantly (*p* < 0.001; *p* < 0.01) reduced the serum glucose in the treatment groups in contrast to the DC (diabetic control) group (Figure 3A). Figure 3B demonstrated that serum insulin level was considerably (*p* < 0.001) reduced in the DC group compared to the NC group. However, treatment of diabetic rats with 10 mg/kg *rhoifolin* (group IV) and 20 mg/kg *rhoifolin* (group V) noticeably (*p* < 0.01) improved the level of serum insulin compared to the DC group. In the DC group, insulin resistance was considerably (*p* < 0.001) high relative to the NC, but a significant (*p* < 0.001; *p* < 0.01) decline was seen in treated groups after treatment with metformin (group III) and 10 mg/kg and 20 mg/kg doses of *rhoifolin*, respectively, compared to the DC group (Figure 3C).

### 2.5. Effect of Rhoifolin on Serum Lipid Levels

Table 2 demonstrates that the diabetic group had substantially (*p* < 0.001) higher total cholesterol (TC), triglycerides (TG), low-density lipoproteins (LDL), and very-low-density lipoprotein (VLDL) levels than the NC group. However, metformin and *rhoifolin* treatment produced a noteworthy (*p* < 0.001; *p* < 0.01) decrease in TC, TG, LDL, and VLDL levels in contrast to the DC group, indicating a hypolipidemic effect. Furthermore, the high-density lipoprotein (HDL) levels of the DC group rats were considerably (*p* < 0.001) more reduced than those of the NC. On the contrary, HDL levels were appreciably (*p* < 0.001; *p* < 0.01) elevated by continuous treatment of diabetic rats (group III, IV, and V) with metformin, at 10 and 20 mg/kg doses of *rhoifolin*, respectively, for 21 days.

### 2.6. Effect of Rhoifolin on Serum Pro-Inflammatory Markers

**Figure 4:**
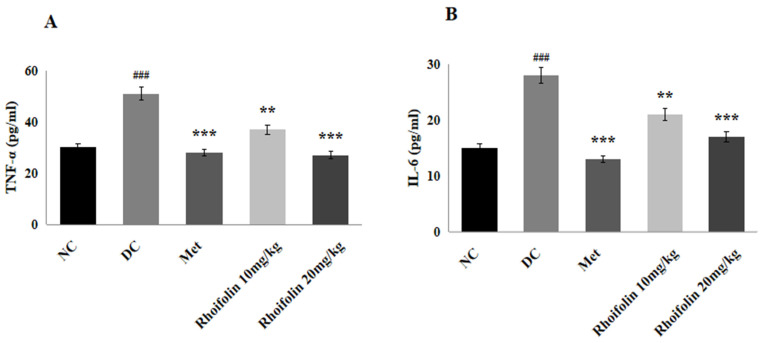
Effect of *rhoifolin* on serum pro-inflammatory markers (**A**) TNF-α; (**B**) IL-6 are presented as Mean ± SD (n = 6/group). ^###^ *p* < 0.001 represents normal vs. diabetic; ** *p* < 0.01, *** *p* < 0.001; diabetic control group vs. treated groups.

Serum levels of pro-inflammatory markers (TNF-α, IL-6) were drastically (*p* < 0.001) more augmented in the DC group than in the NC group. However, the pro-inflammatory cytokine level decreased appreciably (*p* < 0.001) after treatment with metformin and 20 mg/kg of *rhoifolin* compared to the DC group. Treatment of diseased rats of group IV with the lower dose of *rhoifolin* (10 mg/kg) also produced appreciable (*p* < 0.01) results compared to the DC group (Figure 4).

### 2.7. Effect of Rhoifolin on Hepatic Oxidant and Antioxidant Markers

Table 3 demonstrates a considerable (*p* < 0.001) increase in lipid per oxidation (LPO) in diabetic control rats in contrast to the NC group and a marked reduction in LPO following treatment with metformin and *rhoifolin*. A considerable decrease (*p* < 0.001) was observed in SOD, CAT, and GPx levels in the DC group compared to the NC group. However, treatment of diseased rats with *rhoifolin,* particularly with the 20 mg/kg dose for 21 days, resulted in a remarkable (*p* < 0.01) elevation in SOD, CAT, and GPx levels compared to the DC group.

### 2.8. Effect of Rhoifolin on PDX-1 and INS-1 Gene Expression

**Figure 5:**
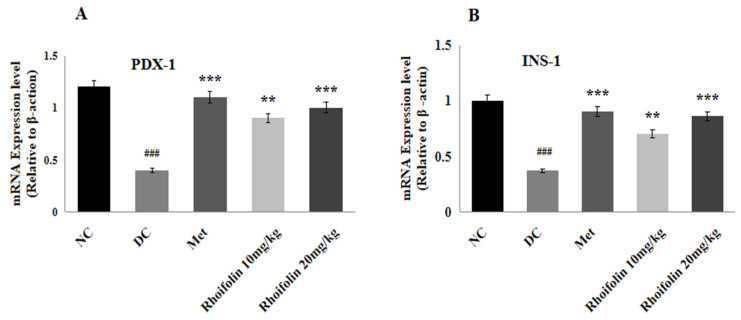
Effect of *rhoifolin* on gene expressions (**A**) PDX-1; (**B**) INS-1 in diabetic rats. Results are displayed as Mean ± SD (n = 6/group). ^###^ *p* < 0.001 represents normal vs. diabetic; ** *p* < 0.01, *** *p* < 0.001; diabetic control group vs. treated groups.

The relative mRNA of PDX-1, and INS-1 involved in the insulin signaling pathway were determined by qRT-PCR using pancreatic tissue. Figure 5A,B represents that streptozotocin considerably (*p* < 0.001) down-regulates the representation of PDX-1 and INS-1 in diabetic control rats. In contrast to the DC group, administration of metformin and *rhoifolin* showed noteworthy (*p* < 0.001*; p* < 0.01) augmentation in PDX-1, and INS-1, expression levels.

### 2.9. Effect of Rhoifolin on SIRT-1 and GLUT-4 Expressions in Pancreatic Tissues

**Figure 6:**
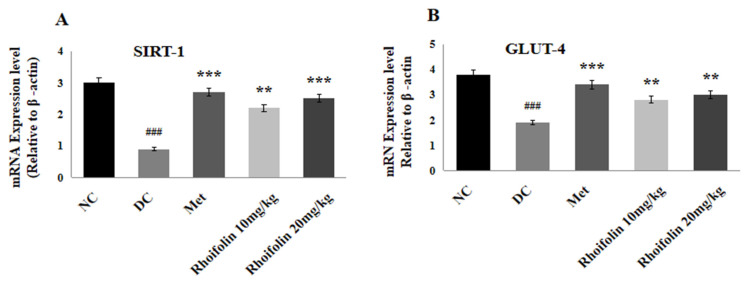
Effect of *rhoifolin* on gene expressions (**A**) SIRT-1; (**B**) GLUT-4 in diabetic rats. Results presented as Mean ± SD (n = 6/group). ^###^ *p* < 0.001 represents normal vs. diabetic; ** *p* < 0.01, *** *p* < 0.001; diabetic control group vs. treated groups.

The expression levels of SIRT-1 and GLUT-4 concerned with insulin secretion and uptake of glucose in liver tissues were also determined by qRT-PCR. Figure 6A,B represents a significant (*p* < 0.001) drop in SIRT-1 and GLUT-4 expression in the DC group compared to normal rats. Administration of 10 and 20 mg/kg of *rhoifolin* led to a noteworthy (*p* < 0.001, *p* < 0.01) increase in SIRT-1 and an appreciable (*p* < 0.01) rise in GLUT-4 expression levels in contrast to the DC group.

### 2.10. Effect of Rhoifolin on JNK Signaling from MAPK Family

**Figure 7:**
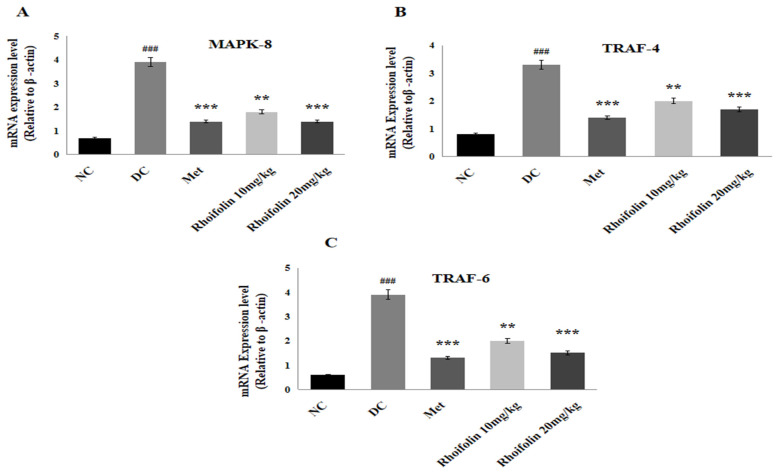
Effect of *rhoifolin* on gene expressions (**A**) MAPK-8; (**B**) TRAF-4; (**C**) TRAF-6 in diabetic rats. Results are showed as Mean ± SD (n = 6/group). ^###^ *p* < 0.001 represents normal vs. diabetic; ** *p* < 0.01, *** *p* < 0.001; diabetic control group vs. treated groups.

Figure 7 shows the effect of *rhoifolin* on the MAPK downstream JNK signaling response in pancreatic tissues of all groups. A significant up-regulation of MAPK-8, TRAF-4, and TRAF-6 expression was viewed in the diabetic control group compared to the normal group. Expression levels of the above-mentioned genes were drastically (*p* < 0.001, *p* < 0.01) down-regulated upon administration of metformin and *rhoifolin*, in contrast to the DC rats.

## 3. Discussion

Diabetes is a chronic metabolic disorder characterized by hyperglycemia due to insufficient insulin production, change in insulin action or a combination of both [19]. Persistent hyperglycemia disrupts the metabolic processes and produces detrimental effects on endocrine, hepatic, renal, cardiovascular and central nervous systems [2,20]. The prevalence of diabetes has grown more quickly in underdeveloped nations [21,22]. According to the World Health Organization (WHO), approximately 80% of the population, particularly in developing countries, relies on plant-based medicines for the treatment of a number of diseases including diabetes [23]. Flavonoids are secondary metabolites of plants and one of the most common families of natural products. In literature, it has been proved that plant flavonoids have potent anti-oxidant and anti-inflammatory activities and play influential roles in the management of chronic diseases such as cardiomyopathy, encephalopathy, diabetes, respiratory, skin, hepatic and renal ailments [24,25]. The phenolic hydroxyl groups present in flavonoids have demonstrated noteworthy anti-inflammatory and anti-oxidant characteristics [26,27]. *Rhoifolin* is a flavanone present in high concentration in bitter orange, bergamot, lemon, tomatoes, bananas, grapes, and grapefruits. Moreover, *rhoifolin* has been found in high concentrations in juices [28].

According to in vitro antioxidant activity, *rhoifolin* showed significant DPPH and ABTS^+^ inhibition, probably due to the ability of *rhoifolin* to scavenge free radicals. Alpha-amylase is a key enzyme of the digestive system, responsible for the hydrolysis of starch into various smaller oligosaccharides which are further broken down into glucose by enzyme α-glucosidase. The resultant glucose is absorbed and enters the bloodstream, ultimately causing postprandial hyperglycemia. Hence, the phytochemicals that have the potential to inhibit these aforesaid two enzymes might be valuable in decreasing the postprandial hyperglycemia-related complications in diabetes mellitus [29,30]. In the present study, *rhoifolin* showed significant in vitro α-amylase inhibitory activity (IC50 = 2.88 mg/mL) and α-glucosidase inhibitory activity (IC50 = 2.7 mg/mL), indicating a hypoglycemic action of *rhoifolin* possibly due to inhibition of these enzymes.

In the present study, hyperglycemia was observed in diabetic rats by using STZ with nicotinamide (NA). As STZ treatment destroys significant numbers of pancreatic β-cells, NA, an antioxidant and form of vitamin B3, in contrast offers partial protection to β cells by inhibiting the harmful effects of STZ, enhancing NAD+ production and scavenging free radicals. Administering NA before STZ results in the loss of early insulin secretion, mimicking the characteristics of T2DM [31]. Glycemic control is the key target for the management of diabetes. In the current investigation, streptozotocin-induced diabetic rats had significantly reduced glucose tolerance, as indicated by increased serum glucose level, reduced insulin secretion and increased insulin resistance, as shown by a high value of Homeostasis Model Assessment of Insulin Resistance (HOMA-IR). In previous studies, it has been documented that STZ triggers insulin resistance and impairs glucose tolerance through the destruction of β-cells in the pancreas [32,33]. Results of in vivo analysis have shown that treatment with *rhoifolin* improved serum glucose levels, HOMA-IR values, and increased insulin secretion, especially at a dose of 20 mg/kg, suggesting an anti-hyperglycemic activity of *rhoifolin*. Possible mechanisms behind anti-hyperglycemic action of *rhoifolin* may include its anti-inflammatory [34], antioxidant properties [35], and ability to enhance β-cell proliferation, increase insulin secretion, decrease β cell apoptosis, and insulin mimetic action [36] by raising insulin sensitivity.

Other anomalies that may emerge from diabetes induced by STZ include lipid abnormality, responsible for increases in TC, TGs, LDL, and VLDL levels. Therefore, it is necessary to manage lipid levels in addition to glucose levels. According to the results, *rhoifolin* exhibits considerable anti-hyperlipidemic properties by decreasing the parameters mentioned above and increasing HDL levels. Oxidative stress associated with damage of beta cells is responsible for reduced insulin secretion and enhanced levels of free fatty acids, which are converted into acetyl CoA in the liver and enhance the triglycerides level. Numerous studies have confirmed that flavonoids have a very effective mechanism for boosting the metabolism of lipid and triglyceride excretion in the feces. According to some research, treatment of STZ-induced diabetic rats with rutin increased the hexokinase and other hepatic enzyme functions involved in the gluconeogenesis and lipid metabolism [37]. Quercetin has also been reported to increase glucose tolerance and hepatic glucokinase activity, as well as reduce hyperlipidemia and hyperglycemia in STZ-diabetic rats [38]. Additionally, morin increased glucose 6 phosphate dehydrogenase (G6PD) and hexokinase activity in STZ diabetic rats [39].

Inflammation and cell damage have a significant relationship that indicates their connection with the etiology of diabetes mellitus. Inflammation occurs due to hyperglycemia-linked oxidative stress. According to the reported results, increased release of pro-inflammatory cytokines led towards degradation of β-cells that are linked to insulin resistance [40]. It has also been observed that administration of *rhoifolin* considerably (*p* < 0.001) decreased the inflammatory markers (TNF-α and IL-6) in diabetic rats. The production of ROS disrupts pancreatic β-cells because of the streptozotocin injection and the ensuing hyperglycemia, which causes diabetes mellitus and complications due to the production of ROS. In the present study, STZ-induced oxidative stress in hepatic tissues resulted in increased LPO levels with concurrent reduced levels of CAT, SOD, and GPx. The treatment with *rhoifolin* restored the antioxidant and oxidant molecules, as shown by increased SOD, CAT, and GPx as well as by reduced levels of LPO in hepatic tissues. The protective action of *rhoifolin* on hepatic tissue is thought to be due to the decrease in oxidative stress and inflammatory markers [41,42]. Flavonoids have therapeutic potential to repair the pancreatic β-cell mass by alleviating inflammation and oxidative stress. According to the findings of this study, *rhoifolin* exerted its antihyperglycemic impact by amplifying insulin sensitivity and secretion. These results are reinforced by previous studies which have shown that flavonoids like luteolin and quercetin potentially enhance the pancreatic islets’ ability to regenerate and maintain normal blood glucose levels in diabetic rats by preventing pancreatic injury and reducing oxidative stress and inflammation [43,44].

The antioxidant defense system and DNA repair processes get overwhelmed by hyperglycemia-induced oxidative stress. It has been demonstrated that a significant part in the pathophysiology of diabetes is played by the activation of the MAPK downstream JNK signaling cascade, which is triggered in response to several stress signals and is involved in cell apoptosis [45,46]. Earlier findings have revealed that pancreatic cells are shielded from oxidative stress damage by the down-regulation of the JNK pathway [41]. According to gene expression analysis, damage of β-cells due to oxidative stress lowers the level of PDX-1, which is an important regulator of the glucose-stimulated insulin gene, and ultimately inhibits the insulin production in diabetic rats. In the current study, PDX-1 and INS-1 expressions were significantly improved in rats treated with *rhoifolin*. These outcomes are consistent with a prior study, according to which treatment of diabetic rats with naringin markedly elevated PDX-1 and insulin gene expression [47]. Another study, which corroborates the findings of the current study, found that a flavonoid called hesperidin increased the expression of PDX-1, which in turn led to increased transcription of the insulin gene [48].

GLUT-4 is a key transporter to uptake the glucose in different tissues and cells. Insulin resistance, release of inflammatory cytokines and production of ROS due to STZ cause a marked decrease in GLUT-4 expression in diabetic rats. However, *rhoifolin* and metformin appreciably up-regulated the expression of GLUT-4 by improving the insulin sensitivity. SIRT-1 is a primary modulator with a critical function in the regulation of the redox balance and lipid/glucose metabolism. SIRT-1 is also responsible for the release of insulin from beta cells in the pancreas and preventing these cells from oxidative stress and inflammation [8]. In the present study, the level of SIRT-1 declined in diabetic rats. However, administration of *rhoifolin* significantly up-regulated the SIRT-1 expression level by improving the antioxidant and inflammatory cytokine levels in treated rats. In previous studies, it is documented that an increased level of SIRT-1 is also associated with an increased concentration of antioxidants (SOD, CAT, GPx) by activating the nuclear factor E2-related factor (Nrf2) [49]. Our study revealed that *rhoifolin* attenuated STZ-induced alterations in diabetic rats by restoring the glycemic control, antioxidant defense markers, inflammatory cytokines, expression levels of JNK signaling and insulin signaling cascade genes.

## 4. Materials and Methods

### 4.1. Experimental Animals and Rhoifolin

Thirty healthy 6–8-week-old Wistar rats (Rattus norvegicus) weighing 180 to 200 g were procured and retained in the animal house at the Institute of Physiology and Pharmacology, UAF, Pakistan. *Rhoifoiln* was purchased from Cayman Chemical, Ann Arbor, MI, USA.

### 4.2. Induction of Disease and Treatment Protocol

The rats were housed for a one-week acclimation period (12 h. light/dark cycle). All animals were maintained on commercial rat chow feed (carbohydrates 42%, proteins 16%, and fats 39%) (manufactured by Fm Browns, Woodrow ave, PA, USA; Model Number 448886). The Institutional Bioethical Committee of the UAF approved all experimental standards and issued an ethical certificate with a registration number (5071/ORIC).

The rats were split up into five groups of six (n = 6) at random. To induce disease, they were fasted for 12 h, and then administered a single IP injection of nicotinamide (120 mg/kg). After 15 min, they received an infusion of 5% dextrose, followed by a single IP dose of STZ (60 mg/kg) [31,50]. Diabetes was confirmed by assessing blood glucose levels through standardized measurement procedures. For the following 21 days, each group received specific food and drug treatments via oral gavage as outlined.

Group I: Normal control (NC), the non-diabetic rats fed a normal diet for 21 days.

Group II: Diabetic control (DC), the STZ-induced diabetic rats fed with a normal diet for 21 days.

Group III: STZ-induced diabetic rats administered with metformin (150 mg/kg) (Catalog Number: M1566 from Spectrum Chemical) as a standard reference drug by oral gavage for 21 days.

Group-IV: STZ-induced diabetic rats treated with *rhoifolin* at a dosage of 10 mg/kg by oral gavage for 21 days.

Group-V: STZ-induced diabetic rats treated with *rhoifolin* at a dosage of 20 mg/kg by oral gavage for 21 days.

### 4.3. Sample Collection

After 21 days of study, animals were starved for 12 h and were euthanized by the introduction of chloroform. A heart puncture was used to obtain blood samples, and serum was separated by centrifuge at 5000× *g* revolutions/minute for 10 min. The serum was then refrigerated at −20 °C for additional biochemical examination. For antioxidant investigations, hepatic tissues were homogenized using buffer solution (Tris-HCl 50 mM and 2 mL of KCl) and centrifuged at 4000× *g* revolutions/minute for 15 min. The supernatant was preserved at −80 °C. For gene expression analysis, hepatic and pancreatic tissues were snap-frozen in liquid nitrogen.

### 4.4. In Vitro Antioxidant Assays

#### 4.4.1. DPPH Radical-Scavenging Assay

The procedure used to calculate *rhoifolin* free radical-scavenging potential was previously documented [51]. *Rhoifolin* was dissolved in methanol to prepare different concentrations (0.1–1.2 mg/mL). After that, 100 μL sample (*rhoifolin*) and 900 μL of DPPH solution were mixed and incubated in darkness. Absorbance at 515 nm was taken by using a spectrophotometer (Thermo Scientific Multiskan; Cat. No. 51119300).

% Inhibition = [(A**_Control_** − A**_Sample_**)/A**_Control_**] × 100

#### 4.4.2. ABTS^+^ Radical-Scavenging Activity

The *rhoifolin* ABTS**^+^** radical-scavenging test was carried out using a 2,2′-azino-bis(3-ethylbenzthiazoline-6-sulphonic acid) ABTS free radical in compliance with a previously published protocol [52]. Dimethyl sulfoxide was used to dilute various *rhoifolin* concentrations to create the sample solution. After mixing 900 μL of ABTS^+^ reagent with 100 μL of the sample solution, the solution was allowed to rest at room temperature for 10 min. Absorbance was measured at 734 nm using Multiskan (Thermo Scientific, Waltham, MA, USA; Cat. No. 51119300) with ascorbic acid as the reference standard. The inhibition percentage was calculated using the following formula.

Radical-scavenging activity (%) = [(A**_Control_** − A**_Sample_**)/A**_Control_**] × 100

### 4.5. In Vitro Alpha-Amylase Assay

An alpha-amylase inhibition assay of *rhoifolin* was accomplished with the previously reported method [53]. *Rhoifolin* was dissolved in DMSO to prepare different concentrations (0.1–1.2 mg/mL). Firstly, 20 μL of each sample of *rhoifolin* was mixed with 20 μL of α-amylase enzyme solution with the addition of 50 μL (0.1 M) PBS and incubated for 20 min. Following that, 100 μL starch (1%) was added. Then, 500 μL of dinitro-salicylic acid reagent (DNSA) was used to terminate the reaction, followed by five minutes in a water bath at 37 °C. The absorbance of the control (acarbose) and sample (*rhoifolin*) solutions was measured at 540 nm using a spectrophotometer (Thermo Scientific Multiskan, Cat. No. 51119300). The α-amylase inhibition of *rhoifolin* was expressed as inhibition.

% Inhibition = [(A**_Control_** − A**_Sample_**)/A**_Control_**] × 100

### 4.6. In Vitro Alpha-Glucosidase Inhibition Activity

A previously described method was performed to assess the alpha-glucosidase inhibitory potential of *rhoifolin* [53]. Potassium phosphate buffer (0.1 M) was used to mix *α*-glucosidase. All the samples were then dissolved in dimethyl sulfoxide. The enzyme solution was then combined with each sample at a volume of 20 mL, and 40 mL of the substrate was incorporated to start the reaction and incubated for 40 min at 37 °C. Then, 80 mL of Na_2_Co_3_ was added to the phosphate buffer to halt the running process. At the end, the quantity of emitted *p*-nitrophenol (pNP) was determined at 405 nm using a Thermo Scientific Multiskan (Cat. No. 51119300) spectrophotometer. The amount of α-glucosidase inhibition of *rhoifolin* and acarbose (control) was determined using the following formula:

% Inhibition = [(A**_Control_** − A**_Sample_**)/A**_Control_**] × 100

### 4.7. Estimation of Fasting Blood Glucose and Determination of Glycemic Parameters

The FBG (Fasting blood glucose) levels were assessed at regular intervals on the 0, 7th, 14th, and 21st day during the trial by using an On-Call Plus (Cat No. G113-11C) glucometer. The glucose and insulin levels in serum were estimated by glucose bioassay kit (GLU1473/3, BioSpain, Barcelona, Spain) and ELISA kit (INS5628, Calbiotect, CA, USA), respectively. The insulin resistance (HOMA-IR) was determined by the method given by [54]:

HOMA-IR=Fasting glucose (mmol/L)×Fasting insulin (μU/mL)/22.5

### 4.8. Estimation of Lipidemic Indicators

The levels of lipidemic markers TC, LDL, HDL, and TG were determined using commercially available kits (TC, Cat. No. MBS168179, MyBioSource; LDL, Cat. No. MBS173145, MyBioSource; HDL, Cat. No. MBS2509097, MyBioSource, TG, Cat. No. MBS2500476, MyBioSource, SanDiego, CA, USA) in accordance with the manufacturer’s instructions.

### 4.9. Estimation of Serum Pro-Inflammatory Markers

The pro-inflammatory biomarkers in serum (TNF-α and IL-6) were determined with ELISA kits (Catalog Number: E-EL-H0109, Elabscience and Catalog Number: E-EL-H0102, Elabscience, Houston, TX, USA) following the instructions of the manufacturers.

### 4.10. Measurement of Hepatic Antioxidant and Oxidant Status

The antioxidant and oxidant statuses were assessed in homogenized hepatic tissues. Glutathione peroxidase (GPx), superoxide dismutase (SOD), and catalase (CAT) were assessed with commercially available ELISA kits (MBS726781,Elabscience; MBS036924, Elabscience and MBS744364; Elabscience, Houston, TX, USA). Lipid peroxidation (LPO) in malondialdehyde (MDA) form was measured using a Thiobarbituric acid reacting substances (TBRAS) colorimetric assay kit (Catalog Number, EEA021; Invitrogen, ThermoFisher Scientific, Waltham, MA, USA).

### 4.11. Real-Time Quantitative PCR for Selected Genes

For the extraction of RNA from pancreatic tissue, the TRIZOL (Thermofisher Scientific, Waltham, MA, USA) technique was employed with some modifications in the previously described method [55]. Nanodrop was used for the quantification of extracted RNA samples. A RevertAid cDNA synthetic kit was used to obtain cDNA. Magnification of cDNA was conceded using Syber Green PCR (Thermo Scientific). The stock solutions for primers were used in agreement with the procedure given by the manufacturer. About 20 µL of the total volume was used for PCR of PDX-1, INS-1, SIRT-1, GLUT-4, MAPK-8, TRAF-4, TRAF-6, and β-actin, along with 2 μL of cDNA pattern, 10 μL of SYBR green, 1 μL of forward and reverse primers into every set of the primers (Table 4, List of primers). The mRNA expressions of runner genes, which were standardized to β-actin, were determined by the 2^−ΔΔCt^ technique.

### 4.12. Statistical Analysis

Data analysis was performed using GraphPad Prism 4.0.1, with results expressed as Mean ± SD for all groups. One-way ANOVA followed by Tukey’s multi-comparison test was performed, with significance set at a 5% level.

## 5. Conclusions

Our findings suggest that *rhoifolin* possess potential anti-diabetic activity via modulation of glycemic control, insulin resistance, lipidemic status, oxidative stress and inflammatory biomarkers. Additionally, the anti-diabetic properties of *rhoifolin* have been shown through its ability to activate genes linked to the insulin signaling pathway while also suppressing the oxidative stress-activated JNK signaling pathway. This research essentially demonstrated the therapeutic significance of *rhoifolin* in the regulation of glycometabolism in the management of diabetes.

## Figures and Tables

**Table 1:** In vitro α-amylase and α-glucosidase inhibitory effects of *rhoifolin*.

α-Amylase Inhibition			α-Glucosidase Inhibition		
Conc. (mg/mL)	% Inhibition by *Rhoifolin*	% Inhibition by Acarbose	Conc. (mg/mL)	% Inhibition by *Rhoifolin*	% Inhibition by Acarbose
0.2	34.5 ± 1.96 ^d^	42.5 ± 0.16 ^d’^	0.2	33.2 ± 0.48 ^d^	40.3 ± 0.73 ^d’^
0.4	43.1 ± 1.27 ^c^	53.7 ± 2.15 ^c’^	0.4	46.7 ± 1.63 ^c^	51.6 ± 1.74 ^c’^
0.6	50.9 ± 2.87 ^b^	61.3 ± 2.74 ^bc’^	0.6	53.6 ± 1.97 ^b^	62.1 ± 2.32 ^b’^
0.8	60.3 ± 3.08 ^ab^	70.6 ± 3.83 ^ab’^	0.8	60.1 ± 2.03 ^ab^*	73.8 ± 3.12 ^ab’^
1.00	65.7 ± 3.26 ^a^*	79.1 ± 4.21 ^a’^	1.00	68.4 ± 2.46 ^a^*	81.5 ± 3.57 ^a’^
IC50 (mg/mL)	0.58	0.35	IC50 (mg/mL)	0.54	0.37

Data represented as Mean ± SD (n = 3). Values with different letters in each column show significant differences (*p* < 0.05) in % inhibitory potency against α-amylase and α-glucosidase between different concentrations (1.00, 0.8, 0.6, 0.4, 0.2 mg/mL) of *rhoifolin* (a–d) and acarbose (a’–d’), respectively. * *p* < 0.05 represents significant differences between % age inhibition by acarbose vs. *rhoifolin.*

**Table 2:** Effect of *rhoifolin* on serum lipid levels.

Groups	Total Cholesterol (mg/dL)	Triglycerides (mg/dL)	High-Density Lipoproteins (mg/dL)	Low-Density Lipoproteins (mg/dL)	Very-Low-Density Lipoproteins (mg/dL)
**Normal control**	76.1 ± 2.16	72.6 ± 3.07	29.9 ± 1.27	32.5 ± 2.02	16.5 ± 1.47
**Diabetic control**	268.6 ± 6.58 ^a###^	287.2 ± 5.75 ^a###^	18.7 ± 1.23 ^a###^	154.7 ± 3.87 ^a###^	42.5 ± 2.84 ^a###^
**Metformin**	104.4 ± 4.67 ^b^***	112.2 ± 4.39 ^b^***	27.4 ± 2.32 ^b^***	54.3 ± 2.68 ^b^***	21.2 ± 1.54 ^b^***
***Rhoifolin*** **(10 mg/kg)**	149.2 ± 5.03 ^b^***	160.6 ± 4.92 ^b^**	20.9 ± 1.24 ^b^**	104.6 ± 3.68 ^b^**	29.3 ± 2.42 ^b^*
***Rhoifolin*** **(20 mg/kg)**	110.1 ± 4.47 ^b^***	128.4 ± 5.31 ^b^***	23.9 ± 1.52 ^b^***	87.03 ± 2.94 ^b^***	24.8 ± 1.94 ^b^***

Results are displayed as Mean ± SD (n = 6/group). ^a###^ significant (*p* < 0.001) difference between normal vs. diabetic control group; ^b^* *p* < 0.05, ^b^** *p* < 0.01, ^b^*** *p* < 0.001; difference between diabetic control group and treated groups.

**Table 3:** Effect of *rhoifolin* on hepatic oxidant and antioxidant markers in diabetic rats.

Groups	TBARS (nmol MDA/mg)	Superoxide Dismutase (U/mg)	Catalase (μmol H_2_O_2_/min/mg)	GPx (nmol/min/mg)
**Normal control**	2.43 ± 0.16	6.5 ± 0.40	54.1 ± 0.62	25.02 ± 0.57
**Diabetic control**	5.53 ± 0.36 ^a###^	3.4 ± 0.36 ^a###^	28.5 ± 0.40 ^a###^	13.03 ± 0.24 ^a###^
**Metformin**	3.20 ± 0.21 ^b^***	5.5 ± 0.40 ^b^**	44.01 ± 2.94 ^b^***	20.2 ± 0.82 ^b^***
***Rhoifolin*** **(10 mg/kg)**	4.76 ± 0.20 ns	3.9 ± 0.09 ns	32.1 ± 2.16 ns	14.96 ± 0.44 ^b^*
***Rhoifolin*** **(20 mg/kg)**	4.13 ± 0.26 ^b^**	4.8 s ± 0.41 ^b^**	39.3 ± 2.05 ^b^**	18.3 ± 0.66 ^b^**

Results are displayed as Mean ± SD (n = 6/group). ^a###^ significant (*p* < 0.001) difference between normal vs. diabetic control group; ^b^* *p* < 0.05, ^b^** *p* < 0.01, ^b^*** *p* < 0.001; noteworthy difference among diabetic control group and treated groups, ^ns^ non-significant difference between diabetic control and treated group.

**Table 4:** List of primers used.

Gene ID	Sequence (5’ to 3’)	Direction
INS-1	ACCCACTGTACCTGGTGTGTG CGGGTCCTCCACTTCACACAC	Forward Reverse
PDX-1	TCGTGAATGGAACCGAGACT TTCATCCACGGGAAAGGGAG	Forward Reverse
SIRT-1	GCAGTAACAGTGACAGTGGC AACTGCCTCTTGATCCCCTC	Forward Reverse
GLUT-4	TTGCCCTTCTGTCCTGAGAG CGCTCTCTTTCCAACTTCCG	Forward Reverse
MAPK-8	GACGACATCGGTCTCTTCG CTGCTGTCAGTATCCGATGC	Forward Reverse
TRAF-6	GCGCCTAGTAAGACAGGACC CACATGCATGCTCTGCGTTT	Forward Reverse
TRAF-4	TCCACACAAGTTCCTGGTGA AGGTGTGGCAGAAGCCGTG	Forward Reverse
β-actin	CGAGTACAACCTTCTTGCAGC TATCGTCATCCATGGCGAACTG	Forward Reverse

## Data Availability

The original contributions presented in this study are included in the article. Further inquiries can be directed to the corresponding authors.
